# Personal

**Published:** 1897-01

**Authors:** 


### Personal.

Dr. P. O. Hooper, of Little Rock, Ark., has been restored to the superintendency of the State Lunatic Asylum, located in that city, from which he was deposed about four years ago through political chicane. It will be gratifying to Dr. Hooper's many personal friends, as well as to everyone interested in the welfare of the insane, that he has been reappointed to this place from which he was so ruthlessly thrust by the merciless hand of politics.

---

Dr. Darwin Colvin, of Clyde, N. Y., chose, Medical expert testimony as the topic of his presidential address to the New York State Medical Association, at its late meeting, October, 1896. Dr. Colvin has presented this subject in an interesting light, and we beg to acknowledge with thanks the receipt of a copy thereof, reprinted from the transactions.

---

Dr. C. C. Fite, of New York, read a paper before the Lycoming County Medical Society at Williamsport, Pa., December 1, 1896, entitled, Antidiphtheritic and antistreptococcic serum: their natural method of production and application for the relief of diseases. This interesting paper is published in full in the *Maryland Medical Journal* of December 19, 1896.

---

Dr. Daniel Lewis and Dr. Charles L. Ogden, of New York, have formed a copartnership and have removed their offices to No. 252 Madison avenue, near 38th street. Dr. Lewis's hours are 10 to 1, and by appointment; Sundays, 10.30 to 1. Dr. Ogden's hours are 8 to 10, 2 to 3 and 5.30 to 7.30, and Sundays by appointment. Telephone 1832, 38th street.

---

Dr. L. Schade, of Buffalo, sailed for Europe in mid-December, for the purpose of paying a visit to his old home at Mecklenburg, Germany. He will be absent but a few weeks.

---

Dr. Max Keiser, of Buffalo, who is pursuing his studies at the General Hospital at Vienna, has been designated as third assistant to Prof. Politzer, a privilege accorded to but few American graduates.

